# Circular Management of *Lavandula stoechas* L. Post-Phytoremediation of Contaminated Soils—From Essential Oil to Potential Biochar for Supercapacitors

**DOI:** 10.3390/life16050716

**Published:** 2026-04-23

**Authors:** María González-Morales, Natalia Díaz-Rodríguez, Luis Francisco Fernández-Pozo, María Ángeles Rodríguez-González

**Affiliations:** 1 Environmental Resources Analysis (ARAM) Research Group, University of Extremadura, 06006 Badajoz, Spain; mariagm@unex.es (M.G.-M.); luferpo@unex.es (L.F.F.-P.); 2 Department of Biochemistry and Molecular Biology and Genetics, University of Extremadura, 06006 Badajoz, Spain; nataliadr@unex.es

**Keywords:** circular economy, phytoremediation, *L. stoechas*, metal(loid)s, distillation

## Abstract

This study deals with a circular economy model to manage biomass of *Lavandula stoechas* L. derived from the phytoremediation of soils with Pb, Zn and Tl metal(oid)s. The species showed high efficacy in phytostabilization, retaining 65% of the metals in the roots. Bioconcentration factors (BAF < 0.5) and translocation (TF < 1) confirmed its behavior as an excluder, minimizing the risk of trophic transfer. This research validated the transformation of this biomass under a zero-residue approach. Via hydrodistillation, essential oils and hydrosols (yield > 0.4%; 0.93 g/mL) were obtained, whose chemical safety was guaranteed by the absence of heavy metals (ICP-MS). Subsequently, the residual biomass was recovered by pyrolysis at 600 °C, obtaining a biochar with a specific surface area (SSA) of 393.7 m^2^/g and an electrical conductivity of 35 S/cm. This performance can be attributed to the synergistic effect of the carbonaceous matrix and encapsulated metals, which act as natural dopants for supercapacitor electrodes. In conclusion, the work demonstrated the transition from hazardous waste to advanced industrial byproducts, integrating environmental remediation with the production of materials for energy storage under safety and sustainability criteria.

## 1. Introduction

Mining activities are a powerful economic and social driver; however, they are also a significant source of waste generation, some of which is hazardous and toxic, as is the case of extraction of metallic minerals, where concentrations of up to 50 ppm can be found [1]. These wastes are the main source of toxicity and environmental pollution of anthropogenic origin [2]. In the European Union, these wastes account for more than 23% of the total waste generated [3].

Studies carried out in abandoned mining areas in the southwest of the Iberian Peninsula [4,5,6,7] have highlighted the severe impact of soil contamination on ecosystem services. As Madouh et al. pointed out [8], the presence of heavy metals in the soil may affect the colonization of certain plant species. However, there are native plants that could develop eco-physiological responses to tolerate the stress caused by such pollution, assimilating heavy metals through their roots and accumulating them in their aerial or root tissues. If these heavy metals enter the food chain [9], detrimental effects on human health and ecosystems can occur [10].

Minimizing heavy metal contamination through phytoremediation techniques (which are environmentally friendly, easy to apply and low cost) is very popular, because it allows the contamination of large land areas to be addressed. Aromatic plants can play a crucial role, as they are inedible and do not facilitate the transfer of heavy metals into the food chain, making them an excellent choice for long-term phytoremediation [11].

One of the most abundant plant species in the Mediterranean biogeographical region is *Lavandula stoechas* L., belonging to the *Lamiaceae* family and *Lavandula genus* [12]. This plant grows as a perennial, aromatic, and branched shrub that can reach up to one meter in height, with violet flowers arranged in spikes. It blooms in spring or early summer with dense and compact inflorescences [13]. Its presence has been described in soils highly contaminated with lead (Pb), zinc (Zn), and thallium (Tl) as a result of mining activities or diffuse pollution from coal combustion in thermal power plants [5]. This aromatic plant contains a large amount of essential oils and other secondary metabolites that make it suitable for gastronomic, cosmetic, and/or medicinal use [14].

The non-volatile compounds present in the essential oil extracted from *L. stoechas* mean these species are considered a source of phytochemicals of pharmacological interest, exhibiting antioxidant, antimicrobial, and anticancer properties, among others [14]. Several ethnobotanical studies have shown the popular uses of some of these species for the treatment of digestion, headaches, heartburn, blood circulation, nasal decongestive bronchitis, and asthma, and as a sedative and antidermatitic [15,16].

With the aim of following the principles of the Circular Green Economy, deep research has been carried out focusing on the use of mining waste and plant biomass in the production of glass [17] and new building materials [18], in wastewater treatment [19], as backfill in infrastructures [20], in bioleaching as a means of mineral extraction [21], or in the encapsulation of toxic elements in cements, preventing their dispersion in the environment and allowing their disposal in controlled landfills or as construction materials after inerting [22]. Other authors stated that both tailings and residual biomass after phytoremediation and/or distillation contain metals with potential for economic revaluation [23] and proposed the conversion of this biomass into carbonaceous materials (biochar) by pyrolysis [24,25] for the synthesis of supercapacitor electrodes.

Supercapacitors are electrochemical energy storage devices that stand out for their ability to deliver a significant energy density without compromising high output power. The success of these systems lies in the use of carbon materials for the manufacture of their electrodes, as they offer high electrical conductivity, corrosion resistance, thermal stability and low production costs. In addition, their high surface area is essential to maximizing the accumulation of electrical charge [26]. However, it has been proven that it is possible to further enhance their capacitance by incorporating multivalent cations from transition metals when generating reversible redox processes [27].

This paper addresses the integral use (zero residue) of the aromatic plant *L. stoechas*.

The selection of *Lavandula* sp. as a biological model is justified by its predominance as a native class in the mining area of this research (an abandoned Pb-Zn mine in the southwest of the Iberian Peninsula), which ensures optimal adaptation to the stress conditions of water and polymetallic soils. However, it also offers an additional advantage since, being an aromatic plant, its biomass allows for the extraction of secondary metabolites (essential oils) and the production of carbonaceous materials (biochar) without competing with crops intended for human consumption, complying with the criteria of safety and industrial sustainability.

This is intended to integrate the principles of the ecological economy with the sustainable management of natural resources, and all with a clear practical applicability within a sustainable business model with a positive environmental and economic impact. Both distillation compounds (essential oil and hydrolate) and biochar could be used in multiple applications.

The proposed application in this study for biochar is its use in supercapacitor electrodes, since there is some research suggesting that metal-contaminated plants after phytoremediation could increase the capacitance (cumulative charge) of supercapacitors [18,28,29].

The controlled presence of these metals does not necessarily represent an impurity. They can work, among others functions, as a functional doping, stabilizing the zinc anode, improving the faradaic efficiency of the storage system, etc. [30,31].

Zinc has been established as a strategic anode material for energy storage due to its low redox potential, stability in aqueous electrolytes and high energy density derived from its two-electron redox reactions [32]. However, the industrial use of zinc hybrid supercapacitors faces critical challenges related to anode reversibility and dendritic structure formation during charge and discharge cycles.

In this context, the incorporation of metal additives such as lead (Pb) and thallium (Tl) plays a fundamental modulating role in morphological control and cyclability. The addition of Pb to the zinc anode is effective in suppressing undesirable morphological changes. Specifically, small amounts of Pb help prevent non-uniform electrodeposition (dendrites) and reduce surface passivation [33]. This mechanism significantly improves the life (cyclability) of the device by ensuring a more stable electrode–electrolyte interface.

Similarly, Tl acts as a modulator of crystalline growth in aqueous electrolytes, optimizing the reversibility of the dissolution/deposition process [34]. In addition, Tl possesses the ability to influence the overpotential of hydrogen evolution, suppressing parasitic reactions of H_2_ generation that often degrade capacitor performance [32,33].

The investigation of this work is innovative because it reports the greatest limitation of conventional phytoremediation when managing contaminated biomass post-remediation, since generally, if it is not treated it ends up in landfills, transferring the environmental problem from one medium (soil) to another (solid waste). To solve this problem, a new challenge is addressed: the circular economy. An economic model is proposed whereby the stabilization of heavy metals is integrated with the synthesis of value-added materials (biochar). In this way, the circular economy exceeds the theoretical concept to consolidate itself as a technical solution and a waste recovery strategy, transforming an environmental responsibility into a functional precursor for energy storage and, effectively, closing the hazardous waste management cycle, with a positive environmental and economic impact.

## 2. Materials and Methods

### 2.1. Phytoremediation Experiment

#### Experimental Design

To evaluate the physiological response of *L. stoechas* to heavy metal stress, a potted trial was performed using a commercial substrate that was enriched with increasing concentrations of Pb (48, 60 and 1500 ppm), Zn (281, 351 and 700 ppm) and Tl (1, 1.25 and 700 ppm). These levels were categorized for each metal as minimum (A), average (B), and maximum (C). The concentrations of Pb and Zn corresponding to level A (48 and 281 ppm, respectively) were established based on the safety thresholds defined by Spanish legislation (BOE-A-2005-895) that guarantee the safety of human health and ecosystems.

In the absence of specific limits in the State legislation for the case of Tl, the Canadian Guidelines for Environmental Quality (CCME, 1964 [35]) were taken as a reference, which establishes the safety limit value at 1 ppm. In accordance with this standard, this concentration was established as minimum level A.

The maximum concentration (C) levels were determined based on the findings reported in a study conducted in an abandoned mining area [4,5], where concentrations of up to 1577 ppm of Pb, 716 ppm of Zn and 744 ppm of Tl were found, values that significantly exceed the maximum permissible concentrations (CMP) of the reference regulations.

The intermediate concentrations present a large difference with respect to the maximum concentrations, but they were prioritized to ensure an exposure gradient that covered both the natural background values/legal threshold (Level A) and the critical levels of extreme toxicity found at the mine site (Level C). Level B was selected to represent moderate–typical contamination observed in the peripheral areas of the mining area [4,5], allowing for evaluation of the response of the plant to a non-linear increase in metal pressure. For elements such as Tl and Pb, this design allows the tolerance threshold of *L. stoechas* to be identified by comparing a controlled exposure (Level B) with a critical exposure of mining soil (Level C), thus evaluating the viability of the species in real remediation scenarios where the distribution of metals is heterogeneous.

To evaluate the growth, accumulation capacity and distribution of heavy metals, an experiment was designed under greenhouse conditions. A uniform batch of commercial *L. stoechas* seedlings (from a certified garden center) was selected to ensure the genetic and phenotypic homogeneity of the initial plant material and to ensure that they all shared an identical crop history (substrate, irrigation, and fertilization) prior to the experiment. Once transferred to the greenhouse, plants were transplanted into pots with the soil under study and kept under a 10-day stabilization period under controlled conditions, guaranteeing an identical starting point for all replicates. Consistency was warranted by selecting individuals with equivalent initial biomass (mean height of 25 cm) and randomly assigning them to treatment groups. Using individuals with the same chronological age and previous nutritional status ensures that the observed differences in growth, physiology, and metal accumulation (Pb, Zn, Tl) are attributable exclusively to experimental treatments (A, B, C) and not to intrinsic variations or previous adaptations of wild material. The final tissue sampling was investigated following a standardized protocol: the roots were washed with a 0.01 M EDTA solution to remove externally adsorbed metals, ensuring that the concentration data reflected only the metal absorbed and translocated by the plant.

Four treatment levels were established (Control, A, B and C) and 32 independent biological replicates were used for each treatment level, which included up to a total of 128 experimental units (32 × 4 levels). The experimental unit consisted of an individual pot with 750 g of substrate and a standardized *L. stoechas* seedling (25 cm in height and 5 roots). The pots (11 cm in diameter and 8 cm in height) were distributed following a Completely Randomized Design (CRD). The assignment of treatments to each unit was carried out using a table of random numbers to ensure the independence of the observations. Since the environmental conditions in the growing area (light, temperature and humidity) were kept strictly homogeneous, a block structure (RCBD) was not necessary. Therefore, the design was analyzed as a single-factor model with four levels of treatment. Pb and Zn were incorporated as nitrates (Pb (NO_3_)_2_, Zn (NO_3_)_2_∙6H_2_O), and Tl as sulphate (Tl_2_SO_4_).

A universal substrate (COMPO SANA^®^, Münster, Germany) was used, where treatments A, B and C were integrated directly into the substrate. According to the data provided by the manufacturer, the chemical characteristics of the substrate used were as follows: slightly acidic pH (5–6.5 in CaCl_2_), nitrogen content as N (200–450 ppm), phosphorus content as P_2_O_3_ (200–500 ppm) and potassium content as K_2_O (300–550 ppm). The analysis of the substrate revealed very low levels of the heavy metal(loid)s studied: Pb (0.01 ppm), Zn (1.60 ppm) and Tl (0.04 ppm).

For environmental control in greenhouses, the temperature was set at 22 °C and the relative humidity at 78%. The irrigation regime was established at three times a week or when the humidity of the substrate fell below 10%. This protocol was designed to simulate the historical rainfall (last 10 years) of the study area (Table 1). Irrigation was carried out with main water early in the morning, with the collection of leachates taking place at the end of the day.

Over a period of two months, biometric growth (length and biomass) of both the aerial and root systems was monitored weekly. Simultaneously, environmental variables and the total concentration of Pb, Zn and Tl in the substrate, leachate and plant tissues were recorded. Physiological parameters (chlorophyll content, photosynthetic rate, and transpiration) were measured every 12 h; for this, a CCM-200 m was used for chlorophyll and an LCi-BioScientific portable system for gas exchange.

All experiments were replicated 3 times with 5 plants from each pot.

### 2.2. Measurement of Metal Content

The metal content (in substrate, plant, and leachate) was determined using ICP-MS with an Agilent Tech model 7900 instrument, Santa Clara, CA, USA. Prior to analysis, solid samples (0.5 g) underwent microwave-assisted acid digestion using a mixture of HNO_3_ and HCl at 180 °C for 20 min. A sample-to-extractant ratio of 1:10 was maintained [30]. Analytical quality was ensured using certified reference materials, with recovery rates ranging between 95% and 105%. Collision cell mode (He) was employed to minimize spectral interferants.

### 2.3. Steam Distillation Experiment

The extraction yield for *L. stoechas* is very low [36], requiring at least 0.5 kg of plant material, but the biomass collected in the pots was not enough. For this reason, 3 kg of *L. stoechas* was collected near the tailings of an abandoned Pb-Zn mine located in the southwest of the Iberian Peninsula, which had been studied in previous works [5]. Once in the laboratory, the thicker stems were removed, and the leaves and flowers were reserved, washed with distilled water, and left to air-dry to remove excess moisture before proceeding with the essential oil extraction.

The distillation was carried out in a 12 L capacity ALBRIGI In Hebra device, Verona, Italy, and each extraction was repeated 3 times. The extraction process was maintained for 60 min. The essential oil separation was carried out in a decanting funnel, and the oil was collected using a capillary pipette. The essential oils were immediately stored in 3 mL amber glass containers and refrigerated at 5 °C. The distilled plants were dried at 60 °C and ground for subsequent chemical analysis by ICP-MS, following the protocol described earlier.

The hydrolat (or floral water) obtained from the distillation was collected and analyzed by ICP-MS to ensure the absence of heavy metals in its composition. The volatile components of the essential oils were determined by gas chromatography coupled to mass spectrometry (GC-MS) using an EVOQ-GCTQ PREM EO&CI system, with 0.5 µL injected.

The effectiveness of the distillation process was evaluated by calculating the yield (the ratio between the mass of the generated product and the plant used) for each experiment [37] using Equation (1).

(1) Yield (%) = (*M*_1_/*M*_2_) × 100

where *M*_1_ is the final mass of essential oil expressed in grams (g) and *M*_2_ is the initial mass of plant material expressed in grams (g).

### 2.4. Statistical Analysis

Statistical analysis was performed using IBM SPSS Statistics software (version 31). Prior to conducting inferential tests, the assumptions of normality, homogeneity of variances, linearity, and homoscedasticity were assessed for the variables at the beginning and end of the trial. Normality was examined using statistical tests and graphical analyses, while homogeneity of variances was assessed using Levene’s test. Linearity and homoscedasticity were checked by inspecting residual plots. The results indicated that none of the variables followed a normal distribution (Table S1, Supplementary Materials).

Consequently, box plots were used to visualize data dispersion, facilitate comparison between groups, and identify potential outliers (Figures S1 and S2, Supplementary Materials). Given the non-parametric nature of the data, the Kruskal–Wallis test was used to detect significant differences between groups. Subsequently, Dunnett’s T3 post hoc test was applied to identify which parameters, under the different heavy metal concentrations, differed significantly from the control group (Tables S2–S6, Supplementary Materials).

The effect size of the comparisons was estimated using Cliff’s δ coefficient, calculated from the Mann–Whitney U statistic for pairwise comparisons. The magnitude of the effect was interpreted according to conventional criteria: negligible (|δ| < 0.147), small (0.147 ≤ |δ| < 0.33), moderate (0.33 ≤ |δ| < 0.474), and large (|δ| ≥ 0.474). Additionally, 95% confidence intervals were calculated for the main descriptive measures and comparisons, providing an estimate of the precision of the results. The level of statistical significance was set at *p* < 0.05.

### 2.5. Biochar Synthesis

The distilled biomass was dried at 100 °C for 24 h and ground to a particle size < 0.5 mm. Pyrolysis was performed in a horizontal tube furnace under a constant flow of nitrogen (100 mL/min) to maintain an inert atmosphere. Samples were heated from room temperature to 600 °C at a heating rate of 5 °C/min, followed by a dwelling time of 4 h. After treatment, the furnace was allowed to cool down to room temperature under nitrogen flow to prevent oxidation. The selection of 600 °C was based on a previous thermogravimetric analysis (TGA), which showed no significant weight loss above this temperature. The yield of the biochar obtained was 68.4%.

### 2.6. Characterization and Instrumentation

The resulting biochar was structurally and texturally characterized to verify its thermal stability and porosity. DTA-TG (TA Instruments analyzer, model Q600, New Castle, DA, USA), infrared spectroscopy (PerkinElmer BXII spectrophotometer, Shelton, CT, USA), Raman spectroscopy (Renishaw inVia spectrophotometer, Gloucestershire, United Kingdom) and nitrogen adsorption–desorption (Micromeritics Tristar 3000, Norcross, GA, USA) were used. Finally, the carbon and nitrogen content was determined by elemental analysis (LECO CS200, LECO TC500, St. Joseph, MI, USA).

## 3. Results and Discussion

### 3.1. Morphometry

**Figure 1:**
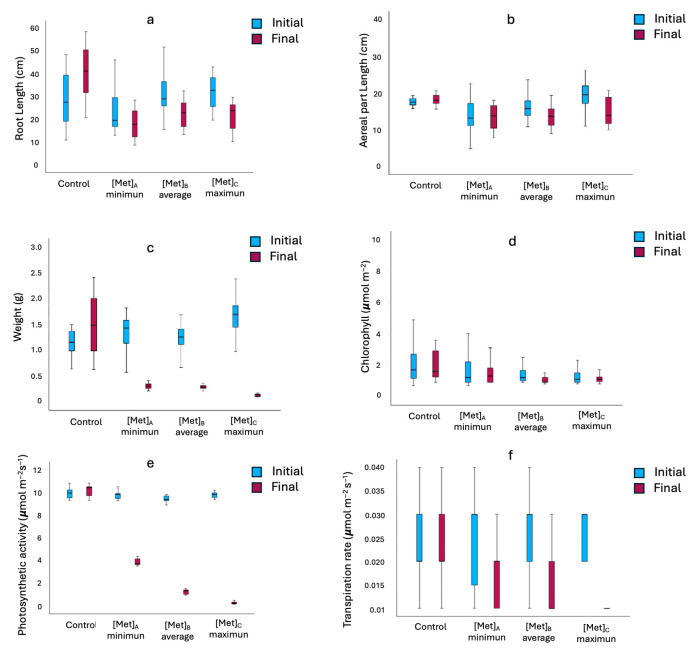
Evolution of the morphological characteristics studied in *L. stoechas* in the presence of metal(loid)s: (**a**) root size, (**b**) aerial part length, (**c**) weight, (**d**) chlorophyl, (**e**) photosynthetic activity and (**f**) transpiration rate.

The degree of damage when exposed to various concentrations of heavy metals (Pb, Zn and Tl) was studied through the evolution during the 8 weeks of the experiment. Figure 1 shows data on plant length (roots and aerial parts), weight and some physiological parameters, such as chlorophyll content, photosynthetic activity and transpiration rate.

The analysis revealed that the exposure to Pb, Zn and Tl inhibited seedling metabolism proportionally to concentration. Growth was affected even at minimal concentrations. However, in treatment A, the reduction in root length was approximately 50%, but the impact on the aerial part was lower (Figure 1a,b). In contrast, treatments B and C showed more severe inhibition, with decreases of up to 80% in total biomass compared to control (Figure 1c). It is important that, despite the high variability in initial and final weight (Table S7), post hoc testing showed no significant differences between treatment levels for dry weight, suggesting that metallic stress impacts growth early and persistently.

Unlike the morphological parameters, the plant physiology showed contrasting responses. For example, photosynthetic activity and transpiration parameters decreased significantly (80% and 60%, respectively) as the metalloid load increased (Figure 1e,f). This reduction in the efficiency of gas exchange explains the lower biomass accumulation observed. However, the chlorophyll content (Figure 1d) remained surprisingly constant (variation of less than 25%), indicating a high resilience of the photosynthetic apparatus of *L. stoechas*.

This chlorophyll stability added to the absence of visual symptoms of wilting or loss of vitality (Tables S7 and S8) and demonstrates the exceptional adaptability of the species to extreme conditions of Pb, Zn and Tl. While authors such as those in Refs. [38,39] reported severe damage to rice seedling physiology from Pb and Cd stress, the present results are more aligned with the findings in Dittrichia viscosa [40], suggesting tolerance mechanisms that prevent irreversible oxidative damage.

The most important changes were in treatment C (maximum concentration), where the greatest losses in photosynthetic activity were observed (*p* < 0.01). The fact that there were no significant differences between treatments B and C in root length (Table S7) indicates that the plant reached a limit of tolerance to moderate–high concentrations of metals. On the other hand, the similarity in the results of treatments A and B (where Pb varied from 48 to 60 ppm) suggests that small increases in this range do not drastically alter the morphology, although any presence of metalloid already represents a decrease with respect to the reference area [5].

It is important to note that the observed resistance in *L. stoechas* was obtained under controlled greenhouse conditions. While the limited volume of soil in the pots could influence the root nature and long-term nutrient availability, this experimental design made it possible to isolate the phytotoxic effect of heavy metals from other environmental stressors. However, extrapolation of these results on a large scale should consider the dynamic variables of real mining soils, where root volume is not restricted and pH can fluctuate seasonally.

### 3.2. Study of Heavy Metal(loid) Content

The results presented in Table 2 confirm that metals were mostly retained in the soil–root system. This preferential accumulation is a characteristic adaptive response of facultative metallophyte plants in the Mediterranean, which act as “excluders” to protect their photosynthetic organs.

In this study, the metal concentrations in the roots were significantly higher than in the aerial part (*p* < 0.01), with a variation in root retention that reached about 20% for Pb and 13% for Zn, with respect to the total absorbed content.

This compartmentalization suggests the existence of active physiological barriers. According to Rahman et al. [38], species of the genus *Lavandula* use adsorption on the cell walls of the root (apoplast) as the first line of defense, in which carboxyl and phosphate groups act as chelators of the divalent cations of Pb^+2^. Other studies in the southwest of the Iberian Peninsula showed a similar phytostabilization strategy for Hg [38] or Zn [39] for *L. pedunculata*.

The marked difference in absorption observed between Pb and Zn, about twofold, is based on their metabolic pathways. Zn is an essential micronutrient absorbed through active metabolic pathways involving specific transporters (e.g., ZIP and AMF families), while Pb is a non-essential element that normally enters the root system through passive, non-metabolic processes, often competing for the same channels as calcium, or by aplastic filtration [39].

On the other hand, Tl accumulates significantly in the root (despite its toxicity), but due to the ionic analogy between Tl^+^ and K^+^, the plant confuses it and is absorbed by the same pathways according to a competition mechanism described by Marjanović et al. [40] by using high-affinity potassium transporters to enter the vascular tissue.

The low bioconcentration factor (BAF < 0.5) and translocation factor (TF < 1) observed in all treatments in this study (Table 3) formally classify *L. stoechas* as a robust excluder species. This low translocation suggests a crucial detoxification mechanism consisting of efficient sequestration in root vacuoles through synthesis of phytochelatins, thus avoiding phytotoxic stress in the photosynthetic apparatus, as suggested by Yang et al., for metallophyte plants under multi-elemental stress conditions [41].

The ability of *L. stoechas* to extract up to 30% of the initial Tl content (Table 2) and act as an efficient biological barrier is surprising. Given the limited information on this metal in the mining ecosystems of Extremadura, the present results are relevant.

The progressive decrease in metals in the leachate at the end of the experiment (in all treatments), with average reductions of 0.2% for Pb and 0.5% for Zn, implies a process of natural attenuation and geochemical retention facilitated by the low acidity of the substrate. In soils with moderate pH, Pb tends to precipitate as secondary mineral phases (e.g., cerussite) or is adsorbed in Fe/Mn oxyhydroxides, minimizing the risk of transport to aquifers [42].

### 3.3. Essential Oil Extraction

The performance parameters of the hydrodistillation process, calculated using Equation (1), are presented in Table 4. The obtained density of the essential oil (EO) was 0.93 g m/L, a value that is within the typical ranges for this species. Although yields were moderate compared to other taxa of the genus *Lavandula* [31], they are consistent with the laboratory standards reported for *L. stoechas* under metal(loid) stress conditions [41]. Recent research [43] suggests that metal stress can induce changes in glandular trichome density, which explains the consistency of our results with standard *L. stoechas* behaviors under stress conditions.

The quality of the essential oil obtained was reflected in its sensory profile, showing great transparency as well as an intensely floral, slightly herbaceous, and quite refreshing aroma. These properties are highly appreciated in aromatherapy applications, as they provide sedative and anxiolytic effects [44].

**Figure 2:**
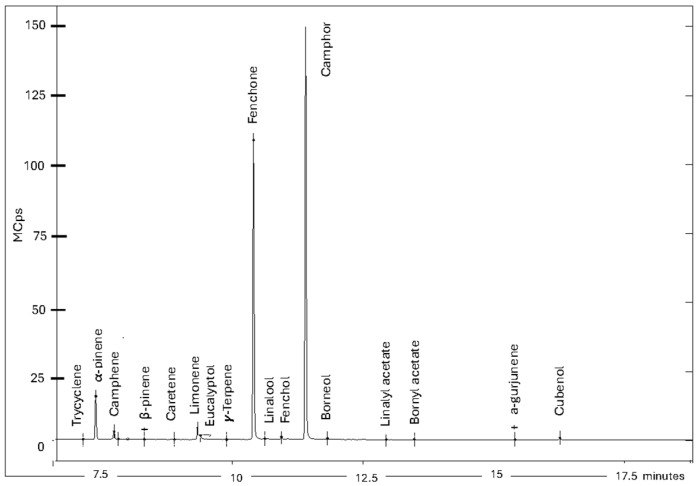
Chromatogram of *L. stoechas* essential oil.

From the phytochemical point of view, more than 40 chemical compounds were identified by gas chromatography (GC-MS), and 20 of them had relative concentrations greater than 1% (Figure 2). The chemical profile was dominated by monoterpenes (α-pinene, limonene), sesquiterpenes (cubenol), ketones (camphor, fenchone), alcohols (linalool, borneol, cubenol), esters (linalyl acetate, bornyl acetate) and oxides (1,8-cineole or eucalyptol). The observed composition is characteristic of Mediterranean chemotypes of *L. stoechas*, where the presence of camphor and 1,8-cineole defines the physicochemical properties and stability of the product [12,45]. This phytochemical profile transcends the taxonomy of the plant, since the abundance of camphor and fenchona not only defines the commercial value of this essential oil in aromatherapy, but also acts as a marker of the plant’s metabolic resilience to Pb and Zn toxicity [46].

A critical aspect for the viability of assisted phytoremediation is the evaluation of the environmental safety of byproducts. The analyses performed by ICP-MS (Table 5) confirmed that heavy metals (Pb, Zn and Tl) were not transferred to the essential oil or hydrolate during the distillation process. This absence of trace metals is explained by the large difference in volatility: while terpenes and aromatic compounds have low boiling points and are carried away by water vapor, metal cations do not have significant vapor pressure at distillation temperatures (100 °C), remaining confined in the post-distillation residual solid biomass [47]. Some recent studies [48] show that the essential oil is inherently safe even in high metallic bioavailability scenarios.

Similar results have recently been documented in other aromatic species grown in degraded soils. For example, studies on *Mentha* sp. and *Ocimum basilicum* have shown that the essential oil matrix acts as a phase free of inorganic contaminants, independently of the metallic load of the soil [49]. In the Iberian Peninsula, research on *L. stoechas* in soils with Hg anomalies also reported no transfer of the metal to essential oil [50], which reinforces the robustness of hydrodistillation as a selective separation method.

After comparing the concentrations of trace metals (Table 5) with the thresholds defined in Chapter 2.4.8 of the European Pharmacopoeia (Ph. Eur.) [51], the levels of Pb, Zn and Tl were determined to be significantly below the permissible exposure limits (PDE), so it can be stated that both the essential oil and the hydrosol (floral water) generated in this study meet the chemical safety criteria for their technical valorization in the cosmetics and fragrance industry.

In conclusion, *L. stoechas* biomass grown in mining areas is not a hazardous waste but rather a high-purity feedstock for the green chemical industry. This allows the cycle of economic recovery of degraded soils to be closed, guaranteeing a risk-free product for the end consumer.

### 3.4. Biochar Characterization

**Figure 3:**
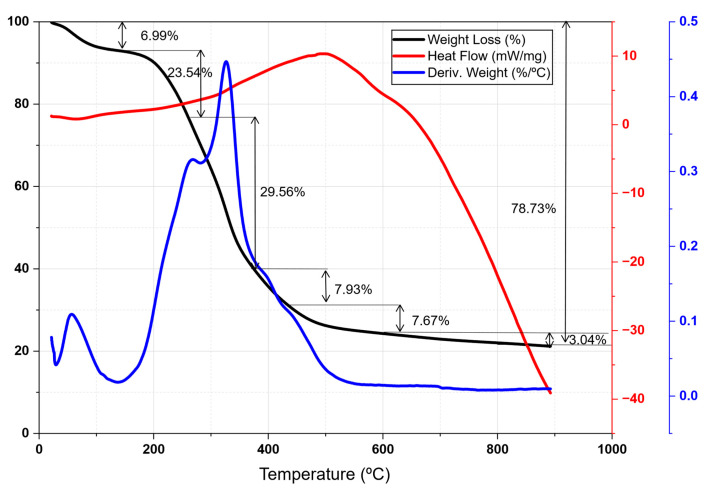
DTA-TG registration of distilled biomass in an inert atmosphere (N_2_).

Figure 3 shows the DTA-TG for the distilled plant. The endothermic peak centered above 80 °C leads to a weight loss of 7% and is attributed to the dehydration of the sample. Afterwards, four stages of weight loss were observed at 270, 360, 430, and 600 °C, corresponding to the thermal decomposition and carbonization of the components of the plant matter: hemicellulose (170–280 °C, 23.54%), cellulose (280–380 °C, 29.56%) and lignin (>380 °C, 18.6%) [52].

#### 3.4.1. Textural Characterization of Biochar (N_2_ Adsorption)

**Figure 4:**
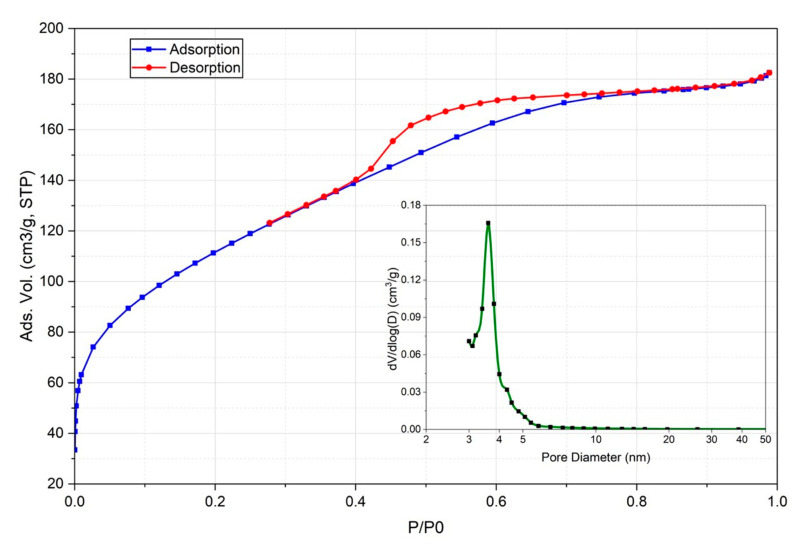
Adsorption–desorption isotherm and pore diameter of the biochar.

The textural characterization of the biochar was carried out by nitrogen physisorption at 77 K. Prior to the analysis, the sample was subjected to a degassing process at 150 °C under nitrogen flux for 18 h to eliminate species adsorbed or occluded in the pores (CO_2_ and water vapor). The resulting adsorption–desorption isotherm (Figure 4) was classified as type IV with a hysteresis cycle, which is characteristic of mesoporous solids according to IUPAC [53]. The observed hysteresis cycle in the relative pressure range P/P0 of 0.4 to 0.8 confirms the presence of mesopores, while the notable increase in the adsorbed volume of nitrogen at low pressures (P/P0 < 0.01) indicates the presence of a network of micropores.

The BET model was used for obtaining the specific surface area (SSA) using the Brunauer–Emmett–Teller (BET) equation [54], applied in the relative pressure range (0.05 ≤ P/P0 ≤ 0.3) where the model presents higher linearity. The biochar presented an SSA of 393.7 m^2^/g.

The pore size distribution of biochar was determined from the desorption curve of the isotherm using the BJH method [55], as is shown in Figure 4. This material presented a trimodal porosity profile, with maximum peaks centered at 3.1, 3.6 and 4.5 nm in diameter. The marked increase in the curve in the region of lower diameters suggests the presence of micropores, while the stabilization of the curve towards larger diameters confirms the absence of significant macroporosity.

By mathematical integration of the pore distribution curve (Figure 4), the contribution of the mesopores to the total SSA was determined to be 158.8 m^2^/g. Since the total SSA was defined as the sum of the meso- and micropore contributions (SSA_BET_ = SSA_micropores_ + SSA_mesopores_), the surface area associated with the micropores was calculated by the difference, resulting in a value of 234.9 m^2^/g. Then, the ratio of micropores to mesopores was approximately 60/40, indicating that biochar had a predominantly microporous nature.

The porous architecture of biochar determines its capacitive properties. In this study, the predominantly microporous structure (<1 nm) is critical to maximizing the charge storage, allowing the electrolyte ions to contribute to high capacitance under moderate current densities [56]. However, the accessibility of these ions can be compromised by increasing the current density, affecting rate performance [57]. On the other hand, the presence of mesopores and macropores (>2 nm) facilitates the fast transport of solvated ions, acting as low-resistance channels [58]. Consequently, although each type of pore plays a specific role, the hierarchical distribution observed in *L. stoechas* biochar balanced a high energy density (provided by micropores) with adequate diffusion kinetics (promoted by mesopores), validating its potential as an electrode in supercapacitors [56].

#### 3.4.2. Thermal Characterization (Atd-Tg)

**Figure 5:**
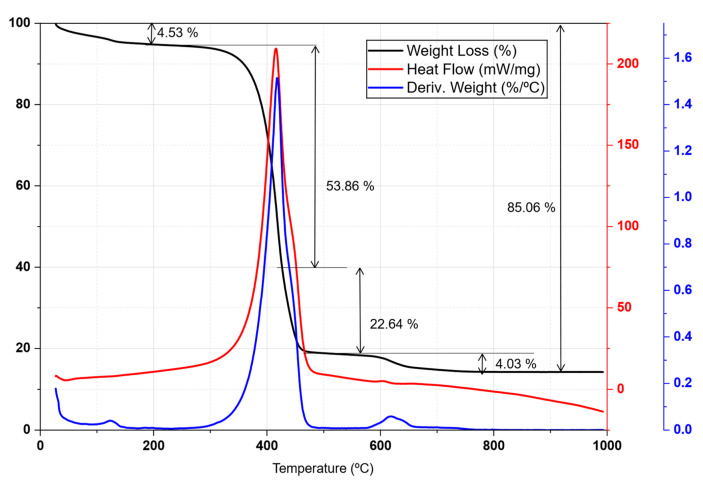
DTA-TG registration of biochar sample in air atmosphere.

The final biochar was also characterized by DTA-TG in air atmosphere to evaluate its thermal stability and quantify the carbon content after pyrolysis, as shown in Figure 5. The DTA curve presented four stages of mass loss. The first stage (20–200 °C, 4.5%) corresponds to the desorption of adsorbed hygroscopic water. The main event occurred between 200 °C and 580 °C, with a 75% loss attributable to carbon combustion. This process was divided into two stages: the oxidation of carbon derived from hemicellulose and cellulose (54.99%) and, subsequently, that of carbon from lignin (20%). These results are consistent with the characterization of the raw material (Figure 3), which reported a hemicellulose and cellulose content close to 50%. Finally, the peak at 640 °C was associated with the combustion of carbon fractions with a higher degree of crystallinity, confirming that the thermal stability of biochar was intrinsically linked to the organic structure of the plant precursor.

#### 3.4.3. Structural Characterization

##### Infrared Spectroscopy

**Figure 6:**
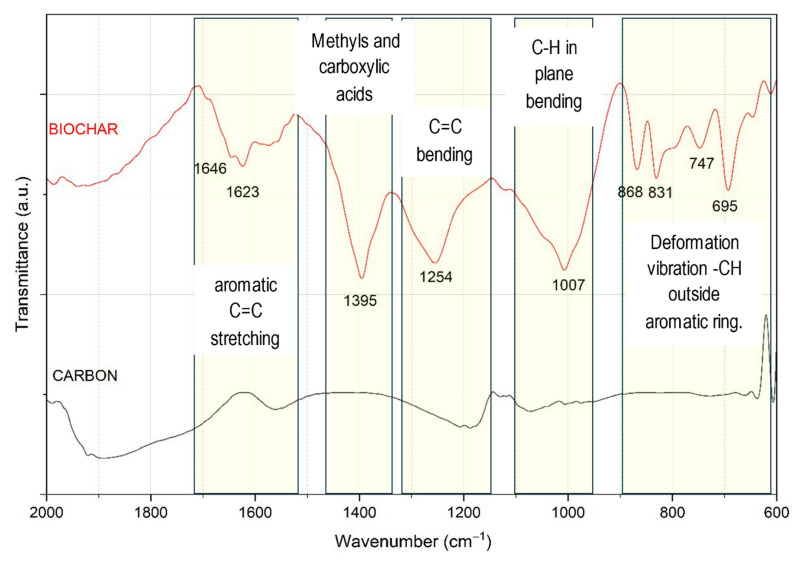
IR spectrum of the biochar obtained and comparison with carbon [60,61,62].

In order to understand the difference between carbon and the obtained biochar, Figure 6 shows the FT-IR (ATR) spectra of both materials. While pure carbon did not show any signals, the presence of vibrational bands in the region of 1500–600 cm^−1^ in the biochar evidenced the existence of components other than elemental carbon. These results confirm the presence of inorganic matter after the pyrolysis process, consistent with the DTA-TG analyses reported by Li & Chen [59].

**Figure 7:**
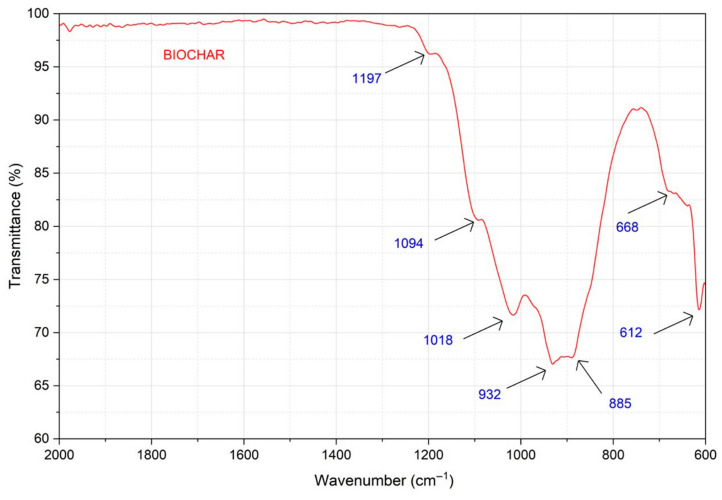
Infrared spectrum of biochar burned at 600 °C.

To quantify the total carbon of the biochar, a combustion process was performed at 600 °C for 4 h. This process transforms carbon into CO_2_ and isolates the inorganic fraction (ash). The analysis of these ashes by IR spectroscopy (Figure 7) revealed characteristic bands of silicon (1094 and 1017 cm^−1^), calcium (885 cm^−1^) and manganese (932 cm^−1^) oxides, results that are consistent with the XRF and XRD analysis shown in Table 6.

##### Raman Spectroscopy

**Figure 8:**
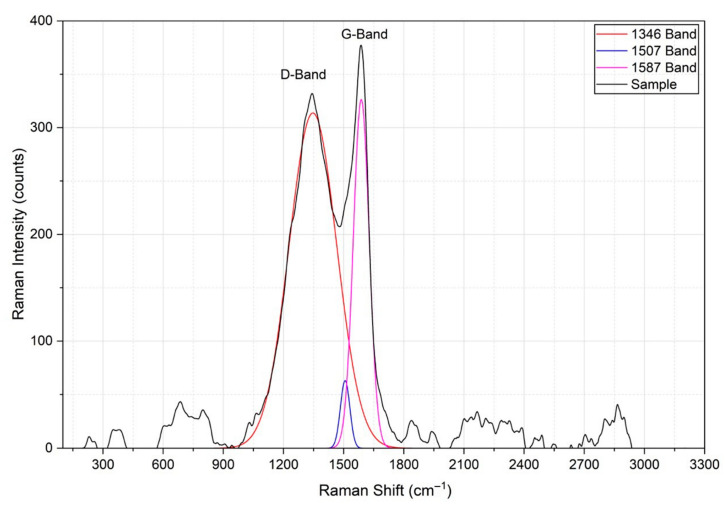
Gaussian deconvolution of the Raman spectrum of biochar.

The Raman spectrum of biochar (Figure 8) presented the distinctive bands of carbonaceous materials. The D band in the 1300–1350 cm^−1^ region and the G band in the 1550–1600 cm^−1^ region, which dominate the Raman spectra of these carbon materials, are directly linked to the vibrational modes of the sp^2^ phase and provide a wealth of structural information. For example, the D band intensity reflects the presence of defects, and the G band correlates with ordered graphitic domains [63].

The ratio of intensities between the D (1350 cm^−1^) and G (1590 cm^−1^) bands, called the ID/IG ratio, is a critical parameter for evaluating the degree of structural disorder and the density of defects in carbonaceous materials. While the G band is associated with the stretching vibrations of sp^2^ bonds in an ideal graphitic structure, the D band is linked to the presence of defects, plane edges, and disorder in the crystal lattice [64].

In terms of structural quality, a reduced ratio is indicative of a graphitic structure of high purity and low defect density. On the contrary, higher values suggest a greater presence of functional groups, structural defects or a greater proportion of basal plane edges. Generally, materials with low crystallinity have ID/IG values in the range of 1 to 2.6, while for highly crystalline carbons this value drops to a range of 0.1 to 0.3 [65].

In the present study, the biochar obtained at 600 °C had an ID/IG ratio = 0.88. This value suggests incipient or moderate crystallinity, characterized by a predominantly disordered structure, but with a tendency towards graphitic organization compared to materials processed at lower temperatures. This hybrid structure is typical of biomass-derived biochars, where small-scale graphitic domains coexist with an amorphous carbon matrix [65].

After the deconvolution of the experimental spectrum, an additional band (D’) was identified at about 1500 cm^−1^. Although its intensity was moderate, this signal confirmed the coexistence of amorphous carbon in the sample (1490 cm^−1^).

##### Chemical Characterization (Elemental)

The obtained biochar of lavender biomass (stems, leaves and flowers) presents a predominantly carbonaceous composition (68.2%). The remaining mass balance (13.4% oxygen and an elemental total of 83.12%) suggests that, together with the organic structural polymers (cellulose, hemicellulose and lignin), inorganic components coexist in the form of metal oxides (Si, Ca, Mn, Ti, etc.). These values are (68% C, 0% S, 1.52% N and 13.4% O).

### 3.5. Application of Biochar in Electrodes

The performance of energy storage systems is conditioned by the properties of their electrodes [66]. However, the disordered structure often exhibited by conventional carbon materials limits electrical conductivity and rarely combines high conductivity with high porosity [67].

Although elemental analysis showed a limited purity (68.2%) and moderate specific surface area (393.7 m^2^/g) compared to activated or graphene-doped carbons [68], the material obtained by pyrolysis of the distilled plant had an acceptable porous structure due to its mesopore and micropore content. It should be noted that, according to Ref. [69], an increase in the specific surface area does not guarantee a greater storage capacity, since the size and distribution of the pores play a more decisive role.

To overcome the limitation of low charge density in supercapacitors, it is common to incorporate polyvalent metal oxides that facilitate faradaic redox reactions, which are the ones that determine the accumulation of electric charge [70]. In this study, biochar already possessed these properties intrinsically since the plant naturally bioaccumulated polyvalent metals (Pb, Zn, Ti, Cu and Fe) in its tissues, as confirmed by the ICP-MS analysis. These elements not only promote charge accumulation but are also probably responsible for the material’s remarkable electrical conductivity (35 S/cm).

The obtained electrical conductivity (35 S/cm) reflects the hybrid nature of biochar, where disordered graphitic domains coexist. Although this value is lower than that of commercial conductive carbons, it is sufficient to ensure the integrity of charge transport in laboratory-scale energy storage devices. Due to the preliminary nature of this research, these results validate the technical feasibility of transforming contaminated biomass into functional electrodes, setting the foundation for future conductivity optimization processes through surface activation treatments. On the other hand, the efficiency of the electrode in supercapacitors does not depend exclusively on intrinsic conductivity, but on the balance between electronic transport, accessible porosity (SSA of 393.7 m^2^/g) and the stability of the electrical double layer [71,72].

## 4. Conclusions

The present study validates *Lavandula stoechas* L. as an effective species for the phytostabilization of multimetallic soils (Pb, Zn and Tl), characterized by an exclusion strategy with a root retention of 65% and translocation factors (TF < 1) that minimize the risk of trophic transfer. However, the dependence of metal mobility and soil pH accentuates the need to manage post-remediation biomass to avoid leaching risks and the reintroduction of contaminants into the ecosystem. The proposed circular economy strategy demonstrated that hydrodistillation is an efficient selective separation method, obtaining essential oils and hydrosols with yields greater than 0.4% and a total absence of heavy metals (confirmed by ICP-MS). This result ensures the chemical safety of these byproducts for recovery in the fragrance and technical cosmetics industry (e.g., soaps), without risk of exposure to inorganic contaminants. Concerning the solid fraction, the post-distillation residual biomass, composed of a lignocellulosic matrix of low thermal stability, reached complete carbonization at 600 °C. The resulting biochar had a specific surface area of 393.7 m^2^/g and a conductivity of 35 S/cm, properties that have been enhanced by the presence of retained metals that act as active centers. Although these properties are preliminary, the material could be optimized by means of surface treatments, which confirms the feasibility of transforming a hazardous waste into a precursor of advanced materials for energy storage.

As a future perspective, it is of great interest to evaluate the effect of the chemical or physical activation of the biochar to increase the porosity and optimize its specific performance in supercapacitors, while investigating the long-term stability of the encapsulated metals in the carbonaceous matrix to ensure zero leaching during the electric charge–discharge cycles. Additionally, it is proposed to explore the capability of intrinsic heavy metals, such as Pb, Zn and Tl, to perform as functional nanocatalysts in organic synthesis processes or in advanced wastewater treatment.

## Figures and Tables

**Table 1:** Average monthly rainfall in the study area over the last 10 years [5].

Month	Jan.	Feb.	Mar.	Apr.	May	Jun.	Jul.	Aug.	Sep.	Oct.	Nov.	Dec.
mL	187	224	268	268	121	41	13	11	121	227	293	211

**Table 2:** Pb, Zn and Tl contents (%) in soil, vegetation and leachate after the study. Treatments: Control; A: minimum metal concentration; B: average metal concentration; C: maximum metal concentration and significance differences (sig) according to the Mann–Whitney U test (different letters in the same column indicate statistically significant differences between groups (*p* < 0.05)).

	**Metal**	**Initial Soil Dosage (ppm)**	**Final Soil (%, sig)**	**Root (%, sig)**	**Aerial (%, sig)**	**Leachate (%, sig)**
Control	Pb	0.01	100	0 b	0	0
	Zn	1.60	25.62	28.12 c	38.12	8.12
	Tl	0.04	25	0 d	75	0.00
A	Pb	48.18	68.34	26.38 e	4.98	0.29
	Zn	281.34	76.80	13.72 f	8.80	0.67
	Tl	1	70	14 a	16	0
B	Pb	60.26	67.59	28.11 g	4.14	0.15
	Zn	352.79	79	14.45 h	0.06	0.57
	Tl	1.25	65.60	21.60 a	12.00	0.80
C	Pb	1500.43	68.13	16.89 i	14.88	0.11
	Zn	706.61	85.92	9.75 k	3.86	0.46
	Tl	700.62	70.37	17.61 l	11.05	0.97

**Table 3:** Bioconcentration and translocation factor. Treatments: A: minimum metal concentration; B: average metal concentration; C: maximum metal concentration.

	**Metal**	**BAF**	**FT**	**Final Soil**	**Root**	**Aerial**
				**(ppm)**		
A	Pb	0.38 ± 0.08	0.19 ± 0.06	32.93 ± 4.25	12.71 ± 1.25	2.40 ± 0.32
	Zn	0.18 ± 0.02	0.64 ± 0.10	216.08 ± 25.30	38.60 ± 4.12	24.76 ± 4.17
	Tl	0.20 ± 0.03	0.94 ± 0.20	0.7 ± 0.06	0.14 ± 0.01	0.16 ± 0.01
B	Pb	0.42 ± 0.12	0.15 ± 0.05	40.73 ± 15.23	16.94 ± 1.23	2.5 ± 0.02
	Zn	0.18 ± 0.06	0.41 ± 0.15	278.72 ± 29.28	51.01 ± 8.84	21.04 ± 1.96
	Tl	0.33 ± 0.08	0.55 ± 0.15	0.82 ± 0.06	0.27 ± 0.01	0.15 ± 0.01
C	Pb	0.25 ± 0.02	0.88 ± 0.20	1022.21 ± 133.87	253.4 ± 33.66	223.22 ± 18.84
	Zn	0.11 ± 0.01	0.39 ± 0.10	607.14 ± 45.23	68.93 ± 9.47	27.26 ± 3.42
	Tl	0.25 ± 0.02	0.63 ± 0.17	493.01 ± 39.39	123.41 ± 9.87	77.42 ± 9.63

**Table 4:** Yield data of the extraction process.

Extraction	Biomass (g)	Essential Oil (mL)	Yield (%)
1	589.88	3.00	0.47
2	434.75	1.96	0.42
3	489.98	2.00	0.38

**Table 5:** Pb and Zn concentration in the soil and plant from the mine, and in the distillation by-products (hydrolat, distilled biomass, distillation wastewater).

	Pb (ppm)	Zn (ppm)	Tl (ppm)
Essential oil	n.d.	n.d.	n.d.
Hydrolat	n.d.	n.d.	n.d.
Biomass after distillation	173 ± 14	307 ± 11	195 ± 15
Residual water after distillation	0.02 ± 0.01	0.96 ±0.04	0.04 ± 0.02
Mine soil	254.6 ± 183.6	367.4 ± 212.4	314.9 ± 95.4
*L. stoechas* mine (leaves and flower)	185.3 ± 92.8	339.8 ± 141.7	216.0 ± 85.1

**Table 6:** Chemical composition of the residue of the burned biochar.

	Al_2_O_3_	SiO_2_	Fe_2_O_3_	CaO	K_2_O	ZnO	SrO	Na_2_O	P_2_O_5_	MgO	TiO_2_	Pb_2_O_3_	MnO	SO_3_
LMA %	6.5	25.8	2.30	31.1	10.3	0.30	0.33	2.41	5.64	10.2	0.36	0.003	0.31	4.26

## Data Availability

The raw data supporting the conclusions of this article will be made available by the authors on request.

## Supplementary Materials

The following supporting information can be downloaded at: https://www.mdpi.com/article/10.3390/life16050716/s1, Table S1. Levene’s test for the morphological. physiological parameters and metal(oid)s concentration. Table S2. Post hoc T3 Dunnett test for the morphological parameters. *: not significance. *p* > 0.05. Table S3. Post hoc T3 Dunnett test for the physiological parameters. *: not significance. *p* > 0.05. Table S4. Post hoc T3 Dunnett test for the metal(oid)s concentration (mg L^−1^) in plants. *: not significance. *p* > 0.05. Table S5. Post hoc T3 Dunnett test for the metal(oid)s concentration (mg L^−1^) in leachate. *: not significance. *p* > 0.05. Table S6. Post hoc T3 Dunnett test for the metal(oid)s concentration (mg L^−1^) in soil. *: not significance. *p* > 0.05. Table S7. Morphological characteristics that showed *p* < 0.01. Note: no significance (NS); significance *p* < 0.05 (*); significance *p* < 0.01 (**). Treatment A: minimum metal concentration; Treatment B: average metal concentration; Treatment C: maximum metal concentration. Figure S1. Box plot concentration of metal(oid)s in plants. Figure S2. Box plot concentration of metal(oid)s in soil and leachate.
