# Supplementary material for: Circular Management of Lavandula stoechas L. Post-Phytoremediation of Contaminated Soils—From Essential Oil to Potential Biochar for Supercapacitors
Source: Life (Basel). 2026 Apr 23;16(5):716. doi: 10.3390/life16050716 (PMC13208435; doi:10.3390/life16050716)

Electronic Supporting Information

**María González-Morales [1]. Natalia Díaz-Rodríguez [2]. Luis Francisco Fernández-Pozo [1, †] . María Ángeles Rodríguez-González [1, †, *]**

[1] Environmental Resources Analysis (ARAM) Research Group. University of Extremadura. 06006 Badajoz. Spain.
[2] Department of Biochemistry and Molecular Biology and Genetics. University of Extremadura. 06006 Badajoz. Spain.
* Correspondence: marodgon@unex.es.
† Authors with equal contribution jointly sharing the first author position.

## TABLE OF CONTENT

Tabla S1. Levene's test for the morphological. physiological parameters and metal(oid)s concentration.

| Test of homogeneity of variances | | | | |
|---|---|---|---|---|
| | Levene's statistic | gl1 | gl2 | Sig. |
| Root length | 9.974 | 3 | 124 | 0.000 |
| Aerial length | 11.267 | 3 | 124 | 0.000 |
| Weight | 72.687 | 3 | 124 | 0.000 |
| Chlorophyll | 3.550 | 3 | 124 | 0.017 |
| Photosynthetic act. | 46.945 | 3 | 124 | 0.000 |
| Transpiration rate | 14.769 | 3 | 124 | 0.000 |
| Zn in root | 30.481 | 3 | 124 | 0.000 |
| Tl in root | 54.280 | 3 | 124 | 0.000 |
| Pb in root | 21.915 | 3 | 124 | 0.000 |
| Zn in aerial | 39.461 | 3 | 124 | 0.000 |
| Tl in aerial | 55.955 | 3 | 124 | 0.000 |
| Pb in aerial | 55.360 | 3 | 124 | 0.000 |
| Zn in leachate | 38.489 | 3 | 124 | 0.000 |
| Tl in leachate | 24.149 | 3 | 124 | 0.000 |
| Pb in leachate | 31.199 | 3 | 124 | 0.000 |
| Zn in soil | 34.060 | 3 | 124 | 0.000 |
| Tl in soil | 55.245 | 3 | 124 | 0.000 |
| Pb in soil | 57.743 | 3 | 124 | 0.000 |

Tabla S2. Post hoc T3 Dunnett test for the morphological parameters. *: not significance. p>0.05.

| **Post hoc T3 Dunnett** | | | | | | | |
|---|---|---|---|---|---|---|---|
| Variable | (I) CONC | (J) CONC | Difference in averages (I-J) | Standard error | Sig. | Confidence interval at 95% | |
| | | | | | | Lower limit | Upper limit |
| Root length (cm) | Control | Minimum | 14.253 | 1.844 | 0.000 | 9.312 | 19.194 |
| | | Medium | 7.995 | 1.853 | 0.000 | 3.031 | 12.959 |
| | | Maximum | 7.737 | 1.958 | 0.001 | 2.502 | 12.973 |
| | Minimum | Medium | -6.258 | 1.377 | 0.000 | -9.936 | -2.580 |
| | | Maximum | -6.516 | 1.515 | 0.000 | -10.564 | -2.467 |
| | Medium | Maximum* | -0.258 | 1.526 | 1.000 | -4.335 | 3.819 |
| Aerial length (cm) | Control | Minimum | 4.052 | 0.477 | 0.000 | 2.766 | 5.337 |
| | | Medium | 3.045 | 0.427 | 0.000 | 1.897 | 4.194 |
| | | Maximum* | 0.600 | 0.600 | 0.896 | -1.019 | 2.219 |
| | Minimum | Medium* | -1.006 | 0.598 | 0.446 | -2.604 | 0.591 |
| | | Maximum | -3.452 | 0.731 | 0.000 | -5.407 | -1.496 |
| | Medium | Maximum | -2.445 | 0.700 | 0.004 | -4.318 | -0.573 |
| Weight (g) | Control | Minimum | 0.480 | 0.096 | 0.000 | 0.225 | 0.735 |
| | | Medium | 0.549 | 0.089 | 0.000 | 0.311 | 0.787 |
| | | Maximum | 0.394 | 0.121 | 0.009 | 0.069 | 0.720 |
| | Minimum | Medium* | 0.069 | 0.096 | 0.978 | -0.188 | 0.325 |
| | | Maximum* | -0.086 | 0.127 | 0.983 | -0.424 | 0.253 |
| | Medium | Maximum* | -0.155 | 0.122 | 0.746 | -0.481 | 0.172 |

Table S3. Post hoc T3 Dunnett test for the physiological parameters. *: not significance. p>0.05.

| Post hoc T3 Dunnett | | | | | | | |
|---|---|---|---|---|---|---|---|
| Chlorophyll (µmol m-²) | Control | Minimum* | 0.467 | 0.187 | 0.082 | -0.035 | 0.969 |
| | | Medium | 0.551 | 0.206 | 0.050 | 0.000 | 1.103 |
| | | Maximum | 0.750 | 0.172 | 0.000 | 0.286 | 1.214 |
| | Minimum | Medium* | 0.084 | 0.165 | 0.996 | -0.356 | 0.525 |
| | | Maximum* | 0.283 | 0.119 | 0.111 | -0.037 | 0.603 |
| | Medium | Maximum* | 0.198 | 0.147 | 0.692 | -0.197 | 0.594 |
| Photosynthetic act. (µmol m-2 s-1) | Control | Minimum | 3.202 | 0.380 | 0.000 | 2.173 | 4.232 |
| | | Medium | 4.658 | 0.518 | 0.000 | 3.253 | 6.063 |
| | | Maximum | 4.975 | 0.601 | 0.000 | 3.347 | 6.604 |
| | Minimum | Medium* | 1.456 | 0.637 | 0.135 | -0.248 | 3.159 |
| | | Maximum* | 1.773 | 0.706 | 0.078 | -0.117 | 3.663 |
| | Medium | Maximum* | 0.317 | 0.789 | 0.999 | -1.790 | 2.425 |
| Transpiration rate (mmol m-2 s-1) | Control | Minimum* | 0.002 | 0.002 | 0.816 | -0.002 | 0.006 |
| | | Medium* | 0.003 | 0.002 | 0.310 | -0.001 | 0.008 |
| | | Maximum | 0.005 | 0.002 | 0.002 | 0.001 | 0.010 |
| | Minimum | Medium* | 0.001 | 0.002 | 0.979 | -0.004 | 0.006 |
| | | Maximum* | 0.004 | 0.002 | 0.161 | -0.001 | 0.008 |
| | Medium | Maximum* | 0.002 | 0.002 | 0.644 | -0.002 | 0.007 |

Table S4. Post hoc T3 Dunnett test for the metal(oid)s concentration (mg $L^{-1}$) in plants. *: not significance. p>0.05.

| Post hoc T3 Dunnett | | | | | | | |
|---|---|---|---|---|---|---|---|
| Variable | (I) CONC | (J) CONC | Difference in averages (I-J) | Standard error | Sig. | Confidence interval at 95% | |
| | | | | | | Lower limit | Upper limit |
| Zn in root | Control | Minimum | -38.154 | 3.259 | 0.000 | -47.276 | -29.032 |
| | | Medium | -50.564 | 1.589 | 0.000 | -55.011 | -46.118 |
| | | Maximum | -68.475 | 1.852 | 0.000 | -73.659 | -63.292 |
| | Minimum | Medium | -12.410 | 3.625 | 0.008 | -22.364 | -2.457 |
| | | Maximum | -30.321 | 3.748 | 0.000 | -40.575 | -20.068 |
| | Medium | Maximum | -17.911 | 2.440 | 0.000 | -24.536 | -11.286 |
| Tl in root | Control | Minimum | -0.134 | 0.006 | 0.000 | -0.151 | -0.117 |
| | | Medium | -0.270 | 0.025 | 0.000 | -0.339 | -0.201 |
| | | Maximum | -123.408 | 2.888 | 0.000 | -131.491 | -115.326 |
| | Minimum | Medium | -0.136 | 0.025 | 0.000 | -0.207 | -0.066 |
| | | Maximum | -123.274 | 2.888 | 0.000 | -131.357 | -115.192 |
| | Medium | Maximum | -123.138 | 2.888 | 0.000 | -131.221 | -115.055 |
| Pb in root | Control | Minimum | -12.708 | 0.844 | 0.000 | -15.069 | -10.347 |
| | | Medium | -16.944 | 0.348 | 0.000 | -17.918 | -15.970 |
| | | Maximum | -253.403 | 13.428 | 0.000 | -290.990 | -215.817 |
| | Minimum | Medium | -4.236 | 0.913 | 0.000 | -6.751 | -1.721 |
| | | Maximum | -240.695 | 13.455 | 0.000 | -278.337 | -203.054 |
| | Medium | Maximum | -236.459 | 13.433 | 0.000 | -274.055 | -198.863 |
| Zn in aerial | Control | Minimum | -24.142 | 2.047 | 0.000 | -29.872 | -18.412 |
| | | Medium | -20.428 | 0.681 | 0.000 | -22.334 | -18.522 |
| | | Maximum | -26.646 | 0.692 | 0.000 | -28.582 | -24.710 |
| | Minimum | Medium* | 3.714 | 2.157 | 0.430 | -2.257 | 9.685 |
| | | Maximum* | -2.504 | 2.161 | 0.814 | -8.483 | 3.474 |
| | Medium | Maximum | -6.218 | 0.970 | 0.000 | -8.851 | -3.586 |
| Tl in aerial | Control | Minimum | -0.132 | 0.007 | 0.000 | -0.150 | -0.115 |
| | | Medium | -0.1191 | 0.010 | 0.000 | -0.146 | -0.093 |
| | | Maximum | -77.391 | 2.017 | 0.000 | -83.037 | -71.745 |
| | Minimum | Medium* | 0.013 | 0.010 | 0.726 | -0.015 | 0.042 |
| | | Maximum | -77.258 | 2.017 | 0.000 | -82.904 | -71.612 |
| | Medium | Maximum | -77.271 | 2.017 | 0.000 | -82.918 | -71.626 |
| Pb in aerial | Control | Minimum | -2.399 | 0.194 | 0.000 | -2.943 | -1.856 |
| | | Medium | -2.502 | 0.098 | 0.000 | -2.778 | -2.227 |
| | | Maximum | -223.224 | 15.067 | 0.000 | -265.397 | -181.050 |

| | | | | | | |
|---|---|---|---|---|---|---|
| Minimum | Medium* | -0.103 | 0.218 | 0.997 | -0.700 | 0.494 |
| | Maximum | -220.824 | 15.068 | 0.000 | -263.001 | -178.648 |
| Medium | Maximum | -220.721 | 15.068 | 0.000 | -262.896 | -178.547 |

Table S5. Post hoc T3 Dunnett test for the metal(oid)s concentration (mg $L^{-1}$) in leachate. *: not significance. p>0.05.

| Post hoc T3 Dunnett | | | | | | | |
|---|---|---|---|---|---|---|---|
| Variable | (I) CONC | (J) CONC | Difference in averages (I-J) | Standard error | Sig. | Confidence interval at 95% | |
| | | | | | | Lower limit | Upper limit |
| Zn in leachate | Control | Minimum | -1.769 | 0.206 | 0.000 | -2.344 | -1.193 |
| | | Medium | -1.884 | 0.064 | 0.000 | -2.063 | -1.706 |
| | | Maximum | -3.152 | 0.093 | 0.000 | -3.412 | -2.892 |
| | Minimum | Medium* | -0.116 | 0.215 | 0.995 | -0.712 | 0.480 |
| | | Maximum | -1.383 | 0.225 | 0.000 | -2.003 | -0.763 |
| | Medium | Maximum | -1.267 | 0.112 | 0.000 | -1.573 | -0.962 |
| Tl in leachate | Control | Minimum | -0.004 | 0.001 | 0.000 | -0.007 | -0.002 |
| | | Medium | -0.014 | 0.001 | 0.000 | -0.018 | -0.010 |
| | | Maximum | -6.779 | 0.129 | 0.000 | -7.139 | -6.418 |
| | Minimum | Medium | -0.009 | 0.002 | 0.000 | -0.014 | -0.005 |
| | | Maximum | -6.774 | 0.129 | 0.000 | -7.135 | -6.414 |
| | Medium | Maximum | -6.765 | 0.129 | 0.000 | -7.125 | -6.405 |
| Pb in leachate | Control | Minimum | -0.138 | 0.016 | 0.000 | -0.184 | -0.093 |
| | | Medium | -0.094 | 0.007 | 0.000 | -0.116 | -0.074 |
| | | Maximum | -1.599 | 0.129 | 0.000 | -1.960 | -1.240 |
| | Minimum | Medium* | 0.044 | 0.018 | 0.105 | -0.006 | 0.093 |
| | | Maximum | -1.461 | 0.130 | 0.000 | -1.824 | -1.099 |
| | Medium | Maximum | -1.505 | 0.129 | 0.000 | -1.866 | -1.145 |

Table S6. Post hoc T3 Dunnett test for the metal(oid)s concentration (mg $L^{-1}$) in soil. *: not significance. p>0.05.

| Post hoc T3 Dunnett | | | | | | | |
|---|---|---|---|---|---|---|---|
| Variable | (I) CONC | (J) CONC | Difference in averages (I-J) | Standard error | Sig. | Confidence interval at 95% | |
| | | | | | | Lower limit | Upper limit |
| Zn | Control | Minimum | -172.271 | 14.487 | 0.000 | -211.558 | -132.983 |
| | | Medium | -212.402 | 17.888 | 0.000 | -260.914 | -163.892 |
| | | Maximum | -402.009 | 39.169 | 0.000 | -508.235 | -295.784 |
| | Minimum | Medium* | -40.132 | 23.018 | 0.404 | -101.655 | 21.392 |
| | | Maximum | -229.738 | 41.762 | 0.000 | -342.269 | -117.208 |
| | Medium | Maximum | -189.607 | 43.060 | 0.000 | -305.375 | -73.839 |
| Tl | Control | Minimum | -172.271 | 14.487 | 0.000 | -211.558 | -132.983 |
| | | Medium | -212.402 | 17.888 | 0.000 | -260.914 | -163.892 |
| | | Maximum | -402.009 | 39.169 | 0.000 | -508.235 | -295.784 |
| | Minimum | Medium* | -40.132 | 23.018 | 0.404 | -101.655 | 21.392 |
| | | Maximum | -229.739 | 41.762 | 0.000 | -342.269 | -117.208 |
| | Medium | Maximum | -189.607 | 43.060 | 0.000 | -305.375 | -73.839 |
| Pb | Control | Minimum | -31.708 | 2.497 | 0.000 | -38.479 | -24.938 |
| | | Medium | -39.895 | 2.731 | 0.000 | -47.301 | -32.490 |
| | | Maximum | -989.319 | 70.854 | 0.000 | -1.181.471 | -797.168 |
| | Minimum | Medium* | -8.187 | 3.700 | 0.159 | -18.071 | 1.698 |
| | | Maximum | -957.611 | 70.897 | 0.000 | -1.149.867 | -765.354 |
| | Medium | Maximum | -949.424 | 70.906 | 0.000 | -1.141.701 | -757.147 |

Table S7. Morphological characteristics that showed p<0,01. Note: no significance (NS); significance p<0,05 (*); significance p<0.01 (**). Treatment A: minimum metal concentration; Treatment B: average metal concentration; Treatment C: maximum metal concentration.

| | Control | A | B | C |
|---|---|---|---|---|
| Root length (cm) | * | * | ** | ** |
| Aerial length (cm) | NS | NS | * | ** |
| Plant weight (g) | * | ** | ** | ** |
| Chlorophyll (µmol $m^{-2}$) | NS | NS | NS | NS |
| Photosynthetic Activity (µmol m-2 s-1) | NS | ** | ** | ** |
| Transpiration Rate (mmol m-2 s-1) | NS | * | ** | ** |

Figure S1. Box plot concentration of metal(oid)s in plants.

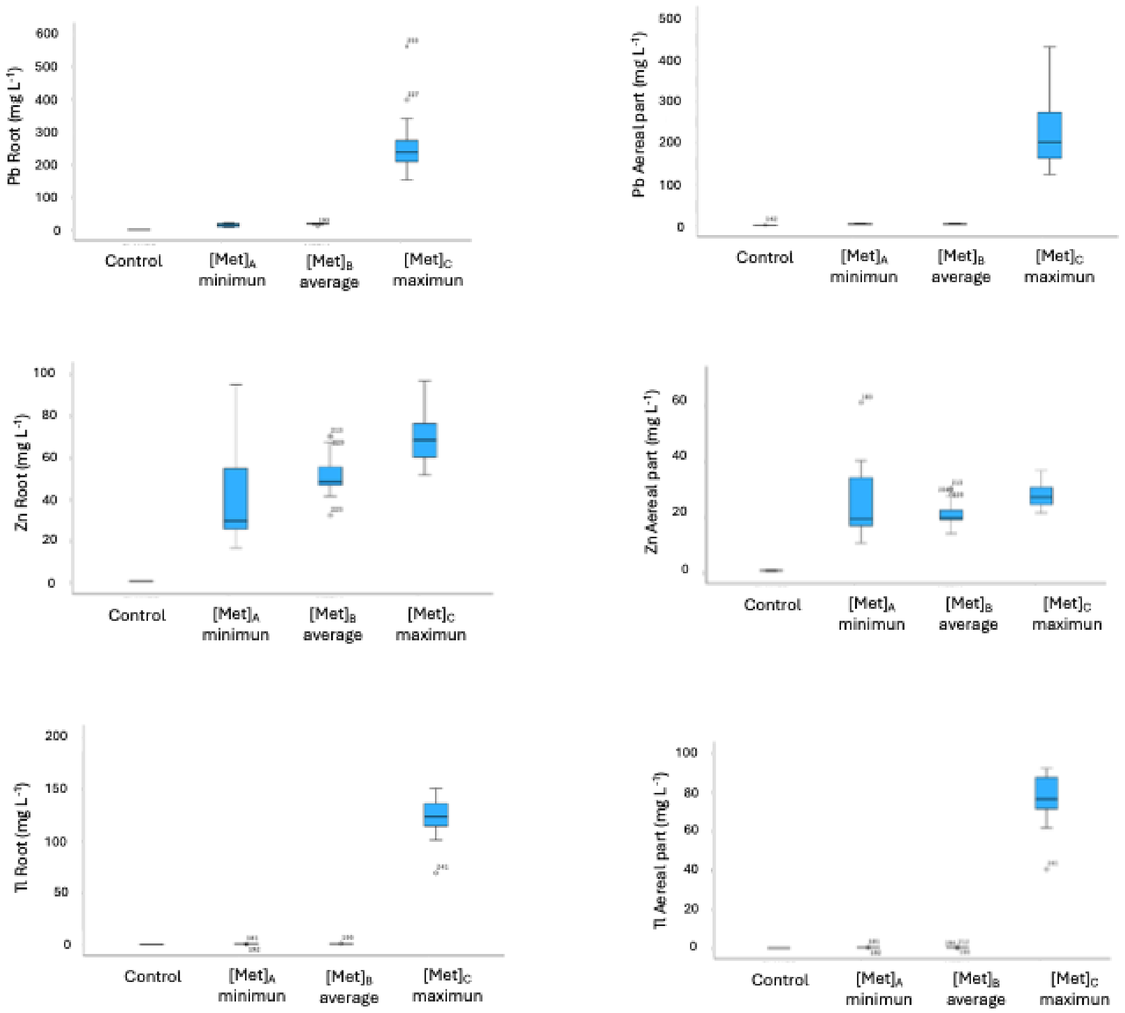

Figure S2. Box plot concentration of metal(oid)s in soil and leachate.

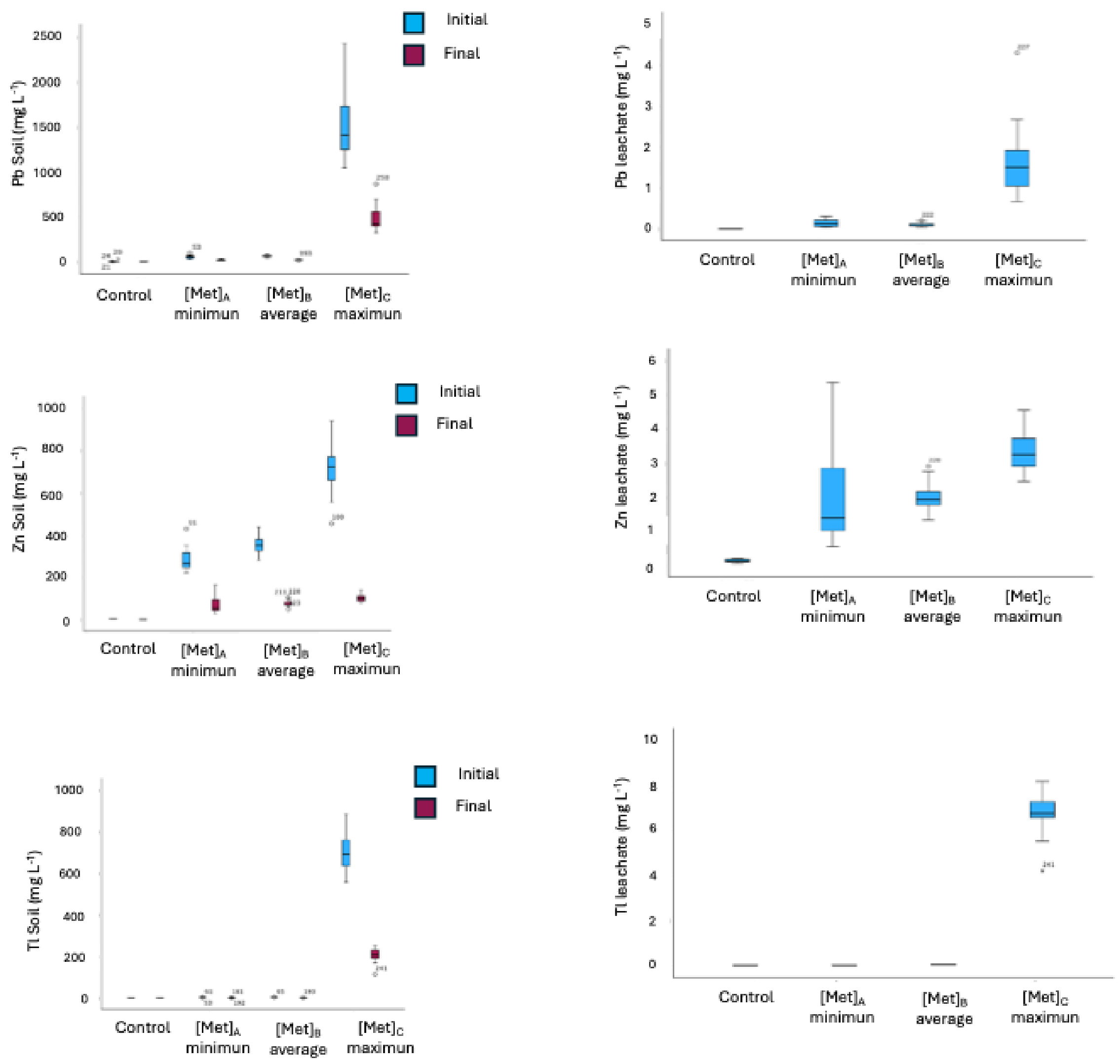

Supplement: Supplementary file 1 [file life-16-00716-s001.zip › Supplementary Lavander Life.pdf]
